# Reconstruction with a free temporoparietal fascia flap for intractable flexor tenosynovitis of the wrist accompanied by a soft tissue defect: a case report

**DOI:** 10.1080/23320885.2021.1986050

**Published:** 2021-11-30

**Authors:** Atsuhiko Iwao, Katsumi Tanaka, Akihito Higashi, Hiroto Saijo, Kazuya Kashiyama

**Affiliations:** Department of Plastic and Reconstructive Surgery, Nagasaki University Hospital

**Keywords:** Flexor tenosynovitis, soft tissue defect, temporoparietal fascia flap

## Abstract

We herein report a case of intractable flexor tenosynovitis. The inflamed synovium was debrided twice because of suspected infectious tenosynovitis. However, it relapsed and caused a soft tissue defect. Reconstruction with a free temporoparietal fascia (TPF) flap was performed. Recurrence has not been detected in the six years after surgery.

## Introduction

Infectious tenosynovitis sometimes requires radical debridement several times [1]; however, soft tissue defects are rare. We herein present a case of intractable wrist flexor tenosynovitis suspected to be caused by nontuberculous mycobacteria (NTM) infection. The flexor tendon and median nerve were exposed due to a soft tissue defect. The defect had to be covered and the gliding surface reconstructed while controlling the infection. Free TPF flap transplantation was very useful in the treatment of this case.

## Case presentation

**Figure 1.**
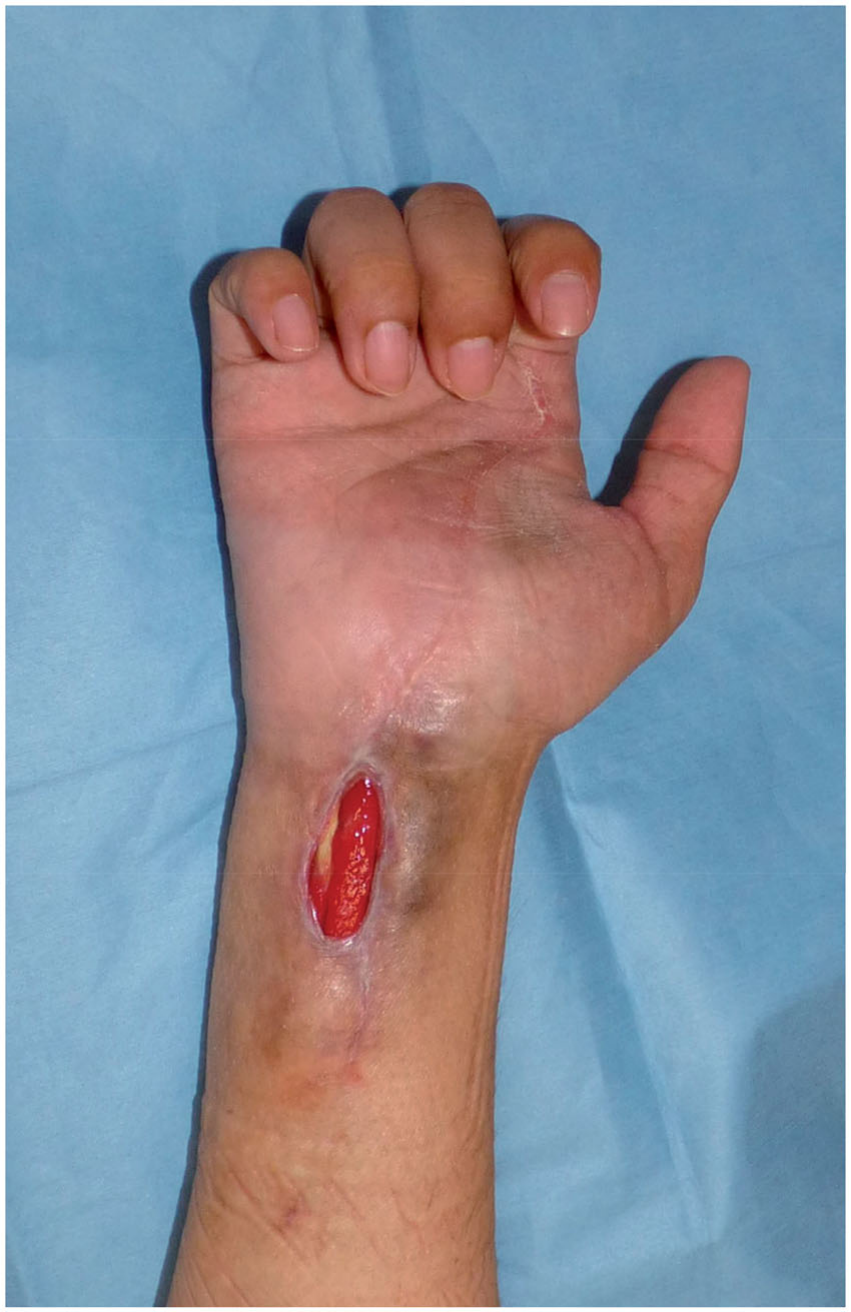
Appearance of the right wrist in the first visit to our outpatient clinic.

**Figure 2.**
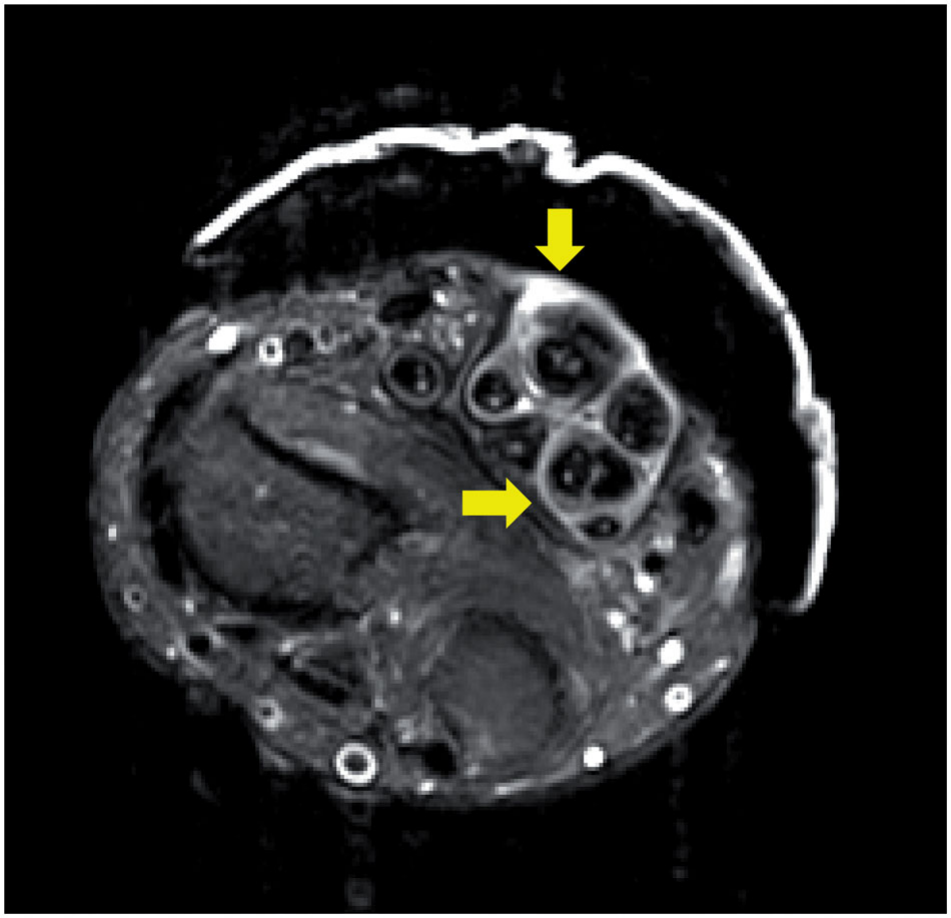
Contrast-enhanced MRI showing hypertrophy of the synovial sheath on the flexor tendon (*arrows*) with T2 fat suppression.

**Figure 3.**
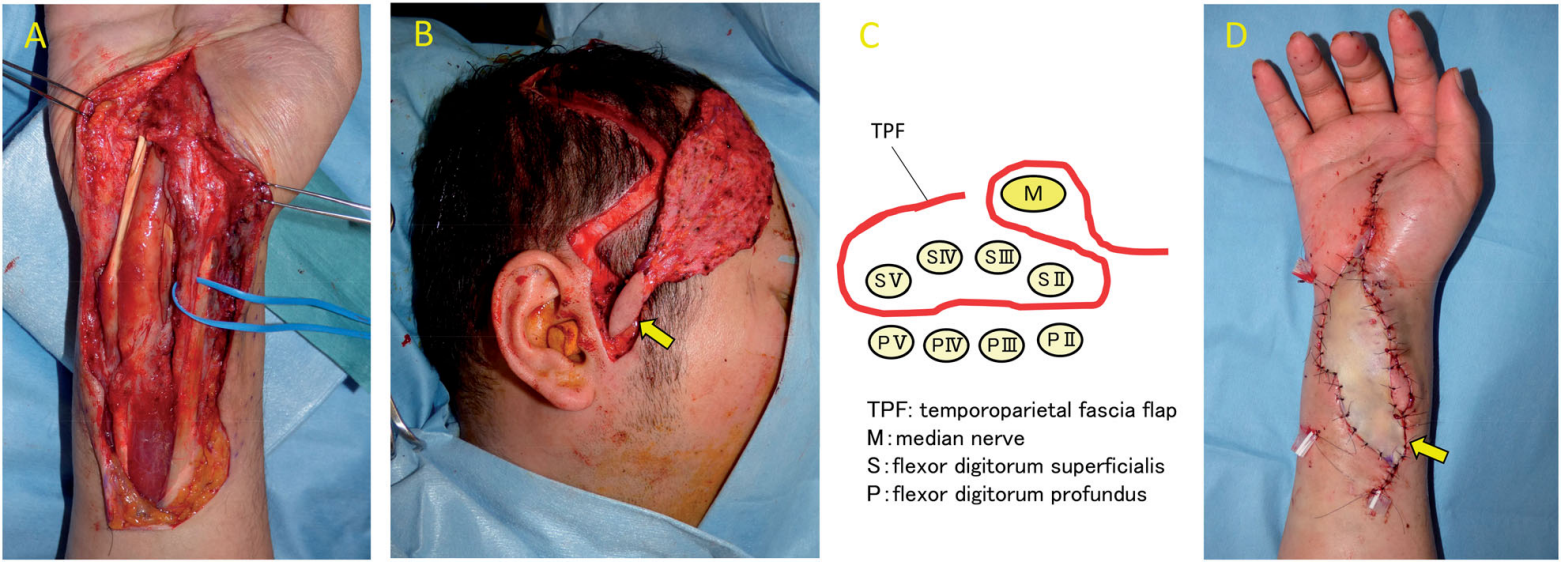
Intraoperative photograph. The wound was filled with an inflamed synovial sheath (**A**). A temporoparietal fascia flap was harvested from the right temporal region. A skin paddle (*arrow*) was made from the anterior part of the right ear (**B**). The median nerve was wrapped in the flap and then placed between FDS and FDP (**C**). A full-thickness skin graft was harvested and grafted onto the flap. *Arrow* indicate anastomosis sight (**D**).

**Figure 4.**
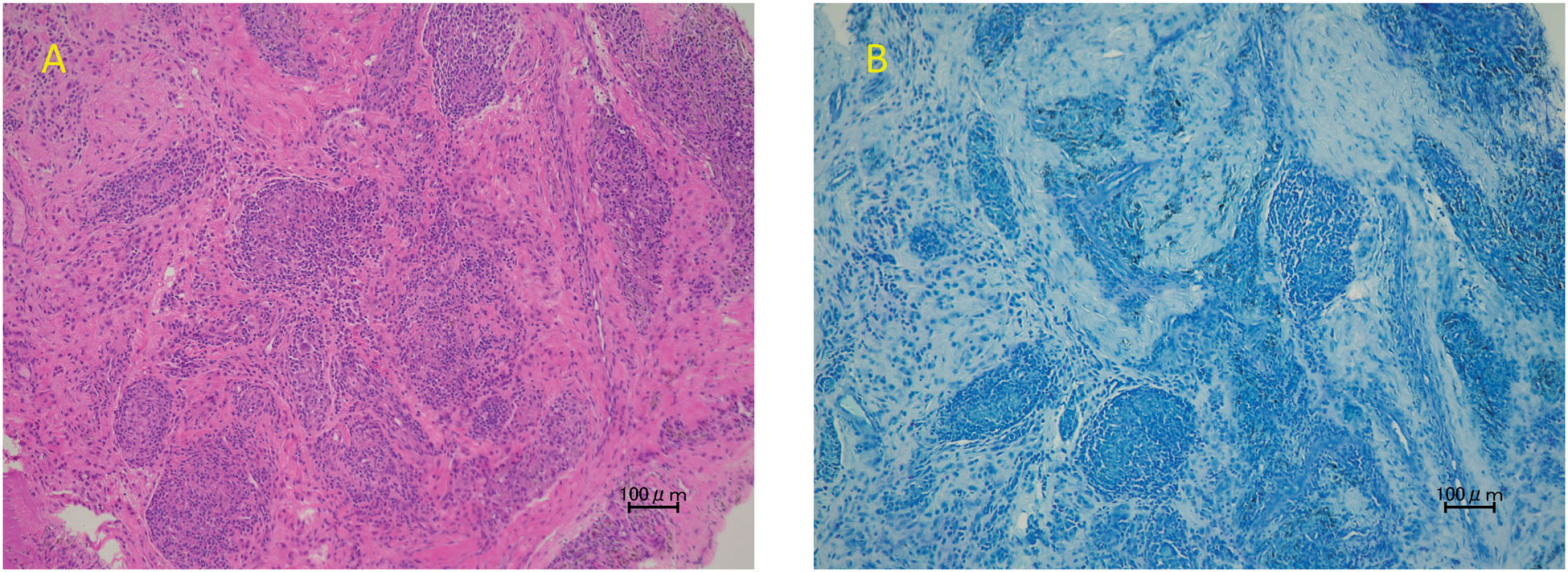
The pathological finding of the synovial sheath with HE staining showed epithelioid cell granuloma (**A**). However, there were no mycobacteria with Thiel-Nielsen staining (**B**).

**Figure 5.**
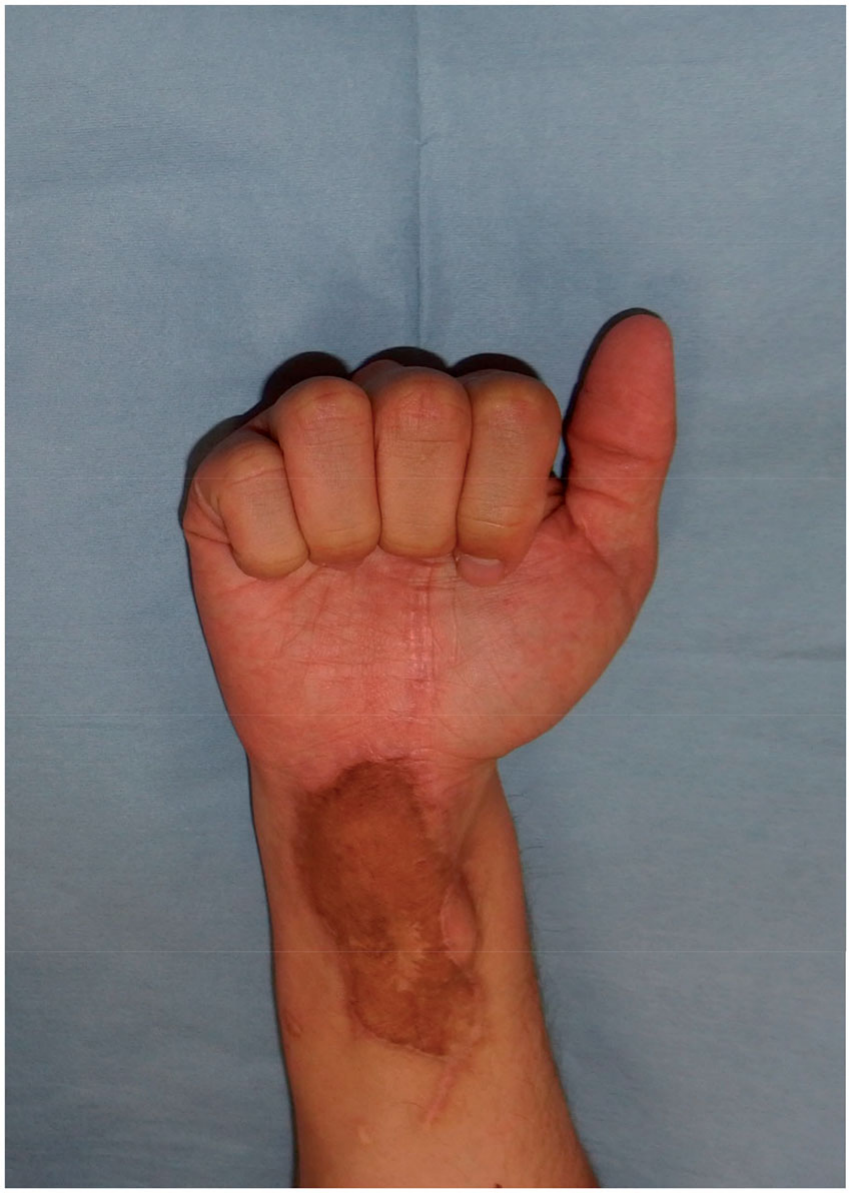
Appearance of the right wrist 1 year and 10 months after surgery.

A 34-year-old man injured his right wrist on the tidal flats. One year later, he visited a local clinic with right wrist pain. Stenosing tenosynovitis was diagnosed and conservative treatment was performed. However, his symptoms persisted for approximately two years after the injury. At another hospital, tenosynovitis with NTM infection was suspected. Therefore, the patient was treated with a combination of radical debridement twice and antibiotics. As for antibiotics, clarithromycin, levofloxacin and rifampicin were administrated for one month from the first surgery to the second surgery. After that, clarithromycin, minocycline, ethambutol and garenoxacin were administrated for five months. However, tenosynovitis did not improve. Six months later, he was referred to our hospital. A physical examination revealed right thenar muscle atrophy and a sensory disorder in the index and middle fingers and on radial side of the ring finger. Semmes-Weinstein monofilament test was 4.56 (red). Range of motion (ROM) was restricted from the index finger to little finger (Table 1). A soft tissue defect was observed on the flexor side of the wrist, which exposed the flexor tendon and median nerve (Figure 1). Laboratory results were normal, except for a C-reactive protein level of 0.1 mg/dL. Magnetic resonance imaging (MRI) revealed hypertrophy of the synovial sheath (Figure 2). The cause was also diagnosed as NTM infection, which was consistent with the previous diagnosis, and was treated with a TPF flap. When the dead space was released during surgery, the wound was filled with an inflamed synovial sheath (Figure 3(A)). The synovium was completely debrided and the carpal tunnel was released. Tenolysis of the flexor tendon and neurolysis of the median nerve were performed. A TPF flap was harvested from the right temporal region (Figure 3(B)). After wrapping the median nerve with the flap, it was inserted between the flexor digitorum superficialis (FDS) tendon and flexor digitorum profundus (FDP) tendon, and then folded onto the FDS tendon (Figure 3(C)). The superficial temporal artery and radial artery were anastomosed end-to-side, then the superficial temporal vein and radial vein were also anastomosed end-to-end. The anastomosis was performed within the lesion, but in a relatively scarless area. A full-thickness skin graft was harvested from the abdomen and grafted onto the flap (Figure 3(D)). Pathological findings of the resected synovium stained with hematoxylin & eosin (HE) revealed epithelioid cell granuloma (Figure 4(A)); however, Ziehl-Neelsen staining did not show any specific findings (Figure 4(B)). Two types of cultures with incubations at 3 and 30 °C, a polymerase chain reaction (PCR) using specific nucleotides for *M. tuberculosis*, *M. intracellulare*, and *M. avium*, and a genetic test targeting other bacteria did not reveal any infection. Antibiotic treatment using clarithromycin and minocycline was performed for 4 months after surgery. The ROM of the right fingers recovered in the six years following surgery (Table 2). The sensory disorder was also recovered to the degree of 2.83 (green) in Semmes Weinstein Monofilament test. No recurrence has been detected (Figure 5).

**Table 1.** Preoperative ROM.

		Extension	Flexion
Thumb	IP	0	50
	MP	0	78
Index finger	MP	0	80
	PIP	−20	70
	DIP	0	42
Middle finger	MP	0	90
	PIP	−5	90
	DIP	0	78
Ring finger	MP	−5	82
	PIP	−5	92
	DIP	0	55
Little finger	MP	0	84
	PIP	−10	54
	DIP	0	60

**TABLE 2.** Postoperative ROM.

		Extension	flexion
Thumb	IP	0	50
	MP	0	78
Index finger	MP	0	84
	PIP	−16	80
	DIP	0	70
Middle finger	MP	0	90
	PIP	0	100
	DIP	0	86
Ring finger	MP	0	90
	PIP	0	96
	DIP	0	80
Little finger	MP	0	92
	PIP	0	90
	DIP	0	86

## Discussion

The free TPF flap was an effective treatment in the present case of intractable tenosynovitis with a soft tissue defect. Limited information is currently available on a simultaneous soft tissue defect and tenosynovitis due to NTM infection. Tun et al. reported that debridement four times for Achilles tendon tenosynovitis due to NTM infection resulted in a soft tissue defect [2]. In the present case, radical debridement conducted twice at the previous hospital caused a soft tissue defect because NTM is an opportunistic pathogen, not a virulent bacterium that destroys tissues. Since the soft tissue around the wrist is thin, repeated debridement may cause defects. Therefore, flap surgery is needed to cover the defect. We selected a TPF flap for the following two reasons. A free tissue transfer was necessary because of the difficulties associated with harvesting a reliable pedicled flap from near the defect. Furthermore, there were concerns regarding the local blood supply in soft tissues due to the spread of inflammation associated with the infection and repeated surgical invasion. The other reason was that reconstructive surgery required a gliding surface and needed to cover the defect. Since a TPF flap is very thin and non-adherent, it is used to reconstruct tendon gliding surfaces in hand surgery. This flap is also highly flexible and, thus, is useful for filling the dead space as a ‘living spacer’ [3]. We harvested the TPF flap including a small area of skin from the anterior ear and used this lesion as a monitor. Since the monitoring flap was located just above the superficial temporal artery, it may reflect the condition of the anastomotic region. This technique appears to be useful because of the difficulties associated with monitoring a temporoparietal fascia flap after skin grafting onto the flap [4]. On the other hand, we must mention the disadvantage of a TPF. The pedicle is so short that vessel anastomosis is sometimes performed intralesionally like this case. Additionally, it is difficult to restore sensation to the area where the TPF has been transplanted. The limitation of the present case was that we did not definitively diagnose NTM infection. The characteristics of this infection, such as inflammation in a physical examination, laboratory results, and radiological imaging, are not distinctive [5]. In addition, it is challenging to detect NTM with a bacterial culture; therefore, false negatives may occur [6]. We also performed a genetic test, but were still unable to detect NTM. However, a histopathological examination for NTM infection, which shows epithelioid cell granulomas in response to long-term foreign bodies, is useful for reaching a diagnosis. Cheung et al. reported that culture results were positive in 40% of cases, whereas pathological findings were positive in 93% [7]. A characteristic history of wound exposure to soil and water will also facilitate a diagnosis [8]. In the present case, NTM infection was diagnosed based on a clinical history of injuries on the tidal flats and the histopathological finding of epithelioid cell granulomas.

## Conclusion

Synovial debridement on the flexor side of the wrist several times may result in a soft tissue defect. The scar bed causes flexor tendon adhesions and median nerve neuropathy. A free temporoparietal fascia flap is therapeutically effective because it fills the defect while preventing re-adhesion.

## References

[1] Moores CD, Grewal R. Radical surgical debridement alone for treatment of carpal tunnel syndrome caused by Mycobacterium Avium complex flexor tenosynovitis: case report. J Hand Surg Am. 2011;36(6):1047–1051. 21571445 10.1016/j.jhsa.2011.03.008

[2] Lui TH, Chan KB. Achilles tendon infection due to Mycobacterium chelonae. J. Foot Ankl Surg. 2014;53(3):350–352. 10.1053/j.jfas.2013.12.025 24529751

[3] Boeckx WD, De Lorenzi F, van der Hulst RRWJ, et al. Free fascia temporalis interpositioning as a treatment for wrist ankylosis. J Reconstr Microsurg. 2002;18(4):269–273. 12022031 10.1055/s-2002-30182

[4] del Pinal F, et al. Outcomes of free adipofascial flaps combined with tenolysis in scarred beds. J. Hand Surg Am. 2014;39:269–279. 24480687 10.1016/j.jhsa.2013.11.030

[5] Yoshida Y, et al. Gouty flexor tenosynovitis of the hand mimicking atypical mycobacterial infection. Mod Rheumatol. 2005;15:427–431. 17029107 10.1007/s10165-005-0428-4

[6] Cheung JP-Y, Fung B, Wong SS-Y, et al. Review article: Mycobacterium marinum infection of the hand and wrist. J Orthop Surg. 2010;18(1):98–103. 10.1177/230949901001800122 20427845

[7] Cheung JPY, Fung B, Ip WY, et al. Mycobacterium marinum infection of the hand and wrist. J Orthop Surg. 2012;20(2):214–218. 10.1177/230949901202000216 22933682

[8] O Falkinham J. 3rd. Environmental sources of nontuberculous mycobacteria. Clin Chest Med. 2015;36(1):35–41. 25676517 10.1016/j.ccm.2014.10.003

